# Comparative Analyses of Dynamic Transcriptome Profile of Heart Highlight the Key Response Genes for Heat Stress in Zhikong Scallop *Chlamys farreri*

**DOI:** 10.3390/antiox13101217

**Published:** 2024-10-10

**Authors:** Xinyuan Wang, Zujing Yang, Cheng Peng, Haitao Yu, Chang Cui, Qiang Xing, Jingjie Hu, Zhenmin Bao, Xiaoting Huang

**Affiliations:** 1 MOE Key Laboratory of Marine Genetics and Breeding, College of Marine Life Sciences, Ocean University of China, Qingdao 266003, China; xywang0521@163.com (X.W.); pengcmiles@163.com (C.P.); haitao0532@foxmail.com (H.Y.); cuichang2020@outlook.com (C.C.); qiangxing@ouc.edu.cn (Q.X.); hujingjie@ouc.edu.cn (J.H.); zmbao@ouc.edu.cn (Z.B.); 2 Laboratory for Marine Fisheries Science and Food Production Processes, Qingdao National Laboratory for Marine Science and Technology, Qingdao 266237, China; 3 Laboratory of Tropical Marine Germplasm Resources and Breeding Engineering, Sanya Oceanographic Institution, Ocean University of China (SOI-OUC), Sanya 572000, China

**Keywords:** *Chlamys farreri*, heart, antioxidant, transcriptome, heat stress

## Abstract

Heat stress resulting from global climate change has been demonstrated to adversely affect growth, development, and reproduction of marine organisms. The Zhikong scallop (*Chlamys farreri*), an important economical mollusk in China, faces increasing risks of summer mortality due to the prolonged heat waves. The heart, responsible for transporting gas and nutrients, is vital in maintaining homeostasis and physiological status in response to environmental changes. In this study, the effect of heat stress on the cardiac function of *C. farreri* was investigated during the continuous 30-day heat stress at 27 °C. The results showed the heart rate of scallops increased due to stress in the initial phase of high temperature exposure, peaking at 12 h, and then gradually recovered, indicating an acclimatization at the end of the experiment. In addition, the levels of catalase (CAT), superoxide dismutase (SOD), and total antioxidant capacity (T-AOC) exhibited an initial increase followed by recovery in response to heat stress. Furthermore, transcriptome analysis of the heart identified 3541 differentially expressed genes (DEGs) in response to heat stress. Subsequent GO and KEGG enrichment analysis showed that these genes were primarily related to signal transduction and oxidative stress, such as the phosphatidylinositol signaling system, regulation of actin cytoskeleton, MAPK signaling pathway, FoxO signaling pathway, etc. In addition, two modules were identified as significant responsive modules according to the weighted gene co-expression network analysis (WGCNA). The upregulation of key enzymes within the base excision repair and gap junction pathways indicated that the heart of *C. farreri* under heat stress enhanced DNA repair and maintained cellular integrity. In addition, the variable expression of essential signaling molecules and cytoskeletal regulators suggested that the heart of *C. farreri* modulated cardiomyocyte contraction, intracellular signaling, and heart rate through complex regulation of phosphorylation and calcium dynamics in response to heat stress. Collectively, this study enhances our understanding of cardiac function and provides novel evidence for unraveling the mechanism underlying the thermal response in mollusks.

## 1. Introduction

The Intergovernmental Panel on Climate Change (IPCC) has declared that the world has been warming since the industrial revolution. The increasing emission of greenhouse gases and the resulting climate change have been identified as the primary drivers of the occurrence of extreme high-temperature weather events, presenting a substantial risk to marine ecosystems [1,2,3,4,5,6,7]. Mollusks, as significant components of marine ecosystems, are ectothermic organisms that exhibit particular sensitivity to fluctuations in temperature [8]. Numerous studies have reported that extreme high temperature has adverse effects on the life stages of mollusks [9,10,11]. For example, mollusks may produce oxygen radicals after exposure to temperature fluctuations, which causes changes in the activity of antioxidant enzymes, including catalase (CAT), superoxide dismutase (SOD), glutathione S-transferases activities (GST), etc. [12,13]. In the study of *Patinopecten yessoensis* and *Haliotis discus hannai*, when exposed to heat stress, the CAT and SOD activities first increased significantly, and then gradually recovered to the control level as the stress time increased [14]. In the Manila clam *Ruditapes philippinarum*, it could enhance the resistance to marine heat waves by decomposing more energy reserves to increase antioxidant capacity and decreasing the energy supply for reproduction [15]. The high activity of SODs in the gills of *Scapharca inaequivalvis* and *Tapes philippinarum* were involved in the physiological role in respiration and protection against oxidative stress caused by environmental fluctuations [16]. These studies indicate that mollusks may possess the capacity to migrate oxidative damage resulting from heat stress and acclimate to climate warming. However, the focus of the aforementioned studies is predominantly on mantle or gill, with limited exploration of the effects on other tissues or organs.

The heart, being a vital component of the circulatory system, is responsible for facilitating gases exchange and transporting substances externally, thereby playing crucial roles in physiology responses of organisms to fluctuations in environmental temperature [15,16,17,18]. In vertebrates, many studies have reported that heart tissue played a crucial functional role in adapting to various external stresses [19,20,21]. Mammalian hearts dynamically adjusted heart rate via the autonomic nervous system, ensuring a seamless transition from resting states to intense physical exertion, thereby accommodating diverse physiological demands [19]. The regulation of blood pressure is identified as a crucial component of the cardiovascular response to stress. Mammalian hearts are noted for their capacity to adjust myocardial contractility, thereby maintaining hemodynamic stability during periods of duress [20]. Morrison and colleagues emphasized the significance of the heart in regulating vascular tone changes, ensuring preferential blood flow to critical organs such as the brain and muscles in the face of adversity [21]. These results highlight the diverse functions of the vertebrate heart in mitigating physiological challenges, encompassing the regulation of heart rate, maintenance of blood pressure, and adjustments of blood distribution, ultimately contributing to the preservation of organismal homeostasis in adverse conditions. In invertebrates, alterations in cardiac function are also responding to environmental changes. For example, the heart rate of mollusks exhibited varying degrees of impact when subjected to high temperature, with instances of cardiac arrest observed at extremely elevated temperatures [22,23,24,25]. In addition, the heart rate of scallop *Argopecten irradians* showed an initial increase followed by a decrease in response to heat stress, which was similar with activity of the antioxidant enzymes CAT and SOD. Further dynamic transcriptome analysis of scallop hearts revealed an active defense mechanism against heat stress during the acute stage (<24 h), including energy supply, correction of misfolded proteins, and enhanced signal transduction [26]. These findings collectively demonstrate the significant role of the heart in facilitating effective adaptation to heat stress. Nevertheless, research on the molecular regulatory mechanisms of the heart in marine invertebrates is currently limited, hindering the understanding of how mollusks adapt to global warming.

Zhikong scallop (*Chlamys farreri*) is an epibenthic and semi-sessile mollusk, which is naturally distributed along the coasts of northern China, Korea, Japan, and Eastern Russia [27]. *C. farreri* is an economically important mollusk in China owing to its large adductor and rich nutrition, which makes a major contribution to aquaculture in China [28]. In recent years, heat stress caused by ocean warming and marine heat waves has brought serious challenges to the growth and reproduction of scallops, even leading to summer mortality under severe conditions [29]. The objective of this study was to investigate the role of the heart in scallops responding to heat stress and potential regulated mechanisms. Firstly, the changes in heart rate and heart amplitude of scallops under heat stress were examined. Subsequently, the dynamic transcriptome response of scallop hearts to heat stress was analyzed. At the same time, the weighted gene co-expression network was constructed to identify the responsive modules and hub genes. Furthermore, the antioxidant activity of scallop hearts was measured to validate their functional role in coping with heat stress. The results will provide new insights into the function of the scallop heart in response to heat stress and contribute to understanding the potential adaption mechanism of scallops to global warming.

## 2. Materials and Methods

### 2.1. Ethics Statement

The experiments in this study were conducted according to the guidelines and regulations established by the Institutional Animal Care and Use Committee (IACUC) of Ocean University of China.

### 2.2. Sample Collection and Heat Stress Experiment

Healthy Zhikong scallops (shell height: 54.95 ± 4.91 mm) were obtained from the scallop farming area in Qingdao (Shandong Province, China) and transported alive to the laboratory. Scallops were cultured in plastic tanks with filtered and aerated seawater (salinity 30.0 psu; pH 8.0; temperature 20.0 °C) for 7 days of acclimation. The seawater was replaced once per day, and scallops were fed daily with *Nitzschia closterium* (1.0 × 10^5^ cells/scallop). After a week of acclimation, a total of 180 scallops were randomly divided into three groups and exposed directly to the seawater at a temperature of 27 °C, which is the upper temperature during summer in the culture area of *C. farreri*. The heart rates of nine randomly selected scallops were detected at nine time points (0 h, 3 h, 6 h, 12 h, 24 h, 3 d, 6 d, 15 d, and 30 d) during heat stress. And the heart tissues of nine scallops were dissected and frozen in liquid nitrogen at the same time points, and they were divided into three groups, with three individuals in each group forming one sample for subsequent RNA extraction and biochemical assay. Among them, the individuals sampled at 20 °C were set as the control group (0 h).

### 2.3. Heart Performance Measurement

Heart performances of scallops were measured by an improved non-invasive monitoring method [30]. In brief, after one week acclimation, scallops were placed in the petri dish (diameter = 110.0 cm, height = 40 cm) for heart performance measurement, and the water temperature (27 °C) was heated and controlled by heating rods-1000W (ChuangNing, Shanghai, China) during measurement. Then, CNY-70 (Newshift, Lisbon, Portugal) optical sensors were fixed with the blue tack (~20 g) to the shell surface above the heart tissues of individuals. Infrared signal variations from CNY-70 were amplified by AMP-03 (Newshift, Lisbon, Portugal), followed via PowerLab 16/35 (ADInstruments, Sydney, Australia), which filtered and transmitted the signal to the computer. Individuals to be tested were placed in seawater, and once the mantle tentacles extended (around 3 min), the instruments were connected for a two-minute measurement. Finally, the software LabChart v8.1.3 (ADInstruments, Sydney, Australia) was applied to record these signals and analyze the heart performance, including heart rate (HR) and heart amplitude (HA).

### 2.4. Oxidative Stress Indexes Determination

Catalase (CAT) activity, superoxide dismutase (SOD) activity, and total antioxidant capacity (T-AOC) of the heart were detected for each sample point (*n* = 3, respectively). The stored heart tissues were mixed with saline solution to create a 10% tissue homogenate for enzyme activity measurement. The CAT, SOD, and T-AOC enzyme activities were determined following the manufacturer’s instructions of commercial kits (Nanjing Jiancheng Bioengineering Institute, Nanjing, China, cat. No. A007-1-1, cat. No. A001-3-2 and cat. No. A015-3-1). The reaction temperature of CAT, SOD, and T-AOC kits are 37 °C. The CAT activity was determined on the basis of the ammonium molybdate method [31]; the SOD activity was measured using the WBT-1 method [32]; and the T-AOC activity was estimated through the FRAP method [33].

### 2.5. RNA Isolation, Library Construction and RNA-Seq Analysis

Total RNA was isolated from the heart of three samples randomly selected at each time point following the protocol described by Hu et al. [34]. The quality of the RNA, including integrity and concentration, was examined by 1.0% agarose gel electrophoresis and a NanoDrop spectrophotometer (Thermo Fisher, Waltham, MA, USA), respectively. A total of 27 RNA-seq libraries were constructed using an RNA-seq library kit (Vazyme, NR603-01, Nanjing, China) and subjected to sequence on Illumina HighSeq 2000 high-throughput platform provided by Novogene Co., Ltd. (Beijing, China).

The raw reads were processed by the FastQC with default parameters [35], and filtered reads were subjected to STAR v2.4.1 [36] to align them with chromosome-level reference *C. farreri* genome [37]. Gene expression profiles were calculated with the form of TPM (Transcripts per Million) [38]. Differentially expressed genes (DGEs) were identified using the edgeR [39] with a statistically significant threshold of |log_2_FC (fold change)| > 1 and *p* value < 0.05.

Gene ontology (GO) enrichment and Kyoto Encyclopedia of Genes and Genomes (KEGG) pathway analysis of DEGs were performed to explore gene function using the R package GOSeq [40] and KOBAS_v2.0 [41], respectively, and *p* value < 0.05 was set as the threshold for significantly enriched.

### 2.6. Quantitative Real Time-PCR Validation

To further validate the reliability of the RNA-seq data, the expression level of five DEGs, including Globin1, Globin2, Transient receptor potential cation channel subfamily A member 1, Heat shock protein beta-1, and Crystallin alpha B, were detected using quantitative real-time PCR validation (qPCR). The Elongation factor 1 alpha, which is widely used as an internal reference gene in bivalves [42,43], was selected as the reference gene. The detailed primers are listed in Table S1. The qPCR was performed using a LightCycler 480 system (Roche, Basel, Switzerland), and the reaction mixture consisted of 1× SYBR Green (Vazyme, Q131-02, Nanjing, China), 0.2 mM primer pairs, and 2 μL of cDNA template in a total volume of 20 μL. The program was as follows: an initial denaturation step (95 °C for 30 s), followed by 35 cycles of denaturing at 95 °C for 5 s and annealing at 60 °C for 20 s. The relative expression level of DGEs was calculated using the 2^−ΔΔCT^ method [44].

### 2.7. Weighted Gene Co-Expression Network Analysis

A weighted gene co-expression network of 27 transcriptomes of the heart was constructed to identify the key co-expressed gene modules connected to various times under heat stress using the R package WGCNA [45]. To make the network with an approximate scale-free topology, the genes that were expressed in all samples were used for network construction, with the following parameters: minimum module size = 200, tree cutting height = 0.99, soft threshold power = 12. To account for multiple tests, false discovery rate (FDR) was calculated using the package WGCNA. The modules with FDR < 0.05 at least in two time points were selected as significant heat responding modules, and genes were connected if the topological overlap between them was more than 0.1. Cytoscape v3.8.0 was used to visualize gene co-expression networks [46].

### 2.8. Data Analysis

Statistical analysis was conducted with one-way analysis of variance followed by post hoc comparison of means-based Turkey’s Least Significant Difference (LSD) test. SPSS Statistics v23 software (IBM Corp, Armonk, NY, USA) was used to determine the differences among different heat stress treatments (*p* value *<* 0.05, *n* = 3).

## 3. Results

### 3.1. Heart Perfomance of C. farreri during Heat Stress

**Figure 1:**
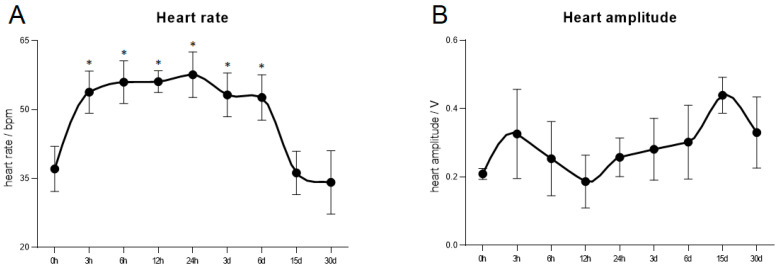
Heart rate and amplitude of *C. farreri* under different heat stress time. (**A**) Heart rate (HR); (**B**) Heart amplitude (HA). The circle shape represents the mean ± S.D. (*n* = 3). (* *p* value *<* 0.05).

As illustrated in Figure 1, with the increase of heat stress time, the heart rate (HR) of *C. farreri* exhibited a trend of initial increase followed by a decrease to control levels. Specifically, the heart rate continuously increased, starting from 3 h, peaking at 24 h with a rate of 57.60 ± 4.94 bpm (beat per minute), then maintaining a relatively high level until a decline commenced after 6 d, eventually returning to control levels after 15 days of stress (Figure 1A). In addition, as time increased, the heart amplitude (HA) reached its first peak of 0.33 ± 0.13 V at 3 h, followed by a continuous decline to a nadir of 0.19 ± 0.08 V at 12 h, and then a subsequent rise to a second peak of 0.44 ± 0.05 V at 15 d, with a slight decrease observed at 30 d. However, there was no significant difference in HA of *C. farreri* during heat stress (*p* > 0.05) (Figure 1B).

### 3.2. Oxidative Stress Indexes

**Figure 2:**
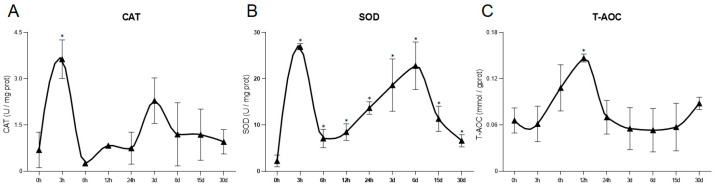
Antioxidant enzymes activity and total antioxidant capacity in heart of *C. farreri* under heat stress. (**A**) CAT activity; (**B**) SOD activity; (**C**) T-AOC. The triangular shape represents the mean ± S.D. (*n* = 3). (* *p* value < 0.05).

The biochemical indexes, CAT, SOD, and T-AOC were used to evaluate the effects of heat stress on oxidative stress in *C. farreri*. The CAT activity initially exhibited a significant increase at 3 h, reaching its peak at 3.63 ± 0.63 U/mg·prot, followed by a rapid decline and subsequent gradual rise at 3 days, ultimately returning to baseline levels (Figure 2A). The SOD activity increased significantly during heat stress, reaching peaks at 26.94 ± 0.66 U/mg·prot and 22.82 ± 5.15 U/mg·prot at 3 h and 6 d, respectively (Figure 2B). T-AOC increased since 3 h, reached a significant peak of 0.15 ± 0.0051 mmol/gprot at 12 h, then rapidly declined to control level (Figure 2C).

### 3.3. Heart Transcriptomes and DEGs Analysis

A total of 1,338,825,848 raw reads were generated with an average of 49.5 M per sample (Table S2). After quality control, the percentage of high-quality reads ranged from 86.41% to 97.65%. High-quality reads were then mapped to the reference genome of *C. farreri* using the STAR, and the ratio of unique mapping of the library was about 71.88% on average. Then the data, combined with gene length information, were utilized to calculate the Transcripts per Million (TPM) values for gene expression.

**Figure 3:**
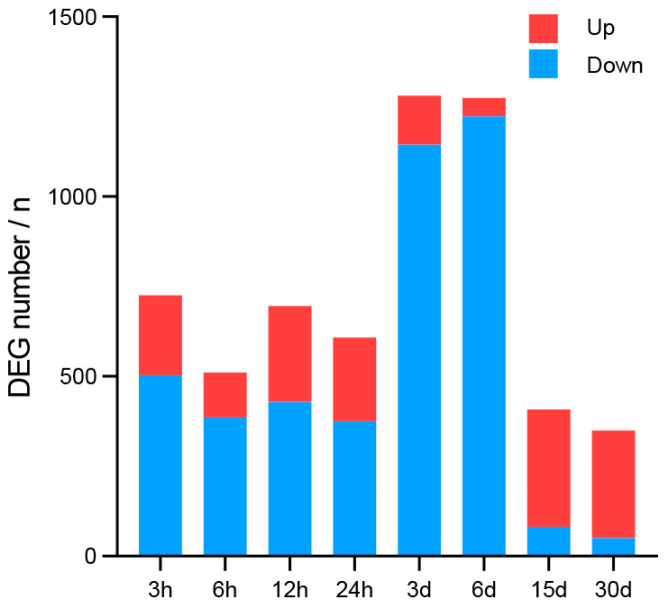
Numbers of DEGs in heart of *C. farreri* under heat stress. Red bar represents the number of up-regulated DEGs; blue bar represents the number of down-regulated DEGs.

**Figure 4:**
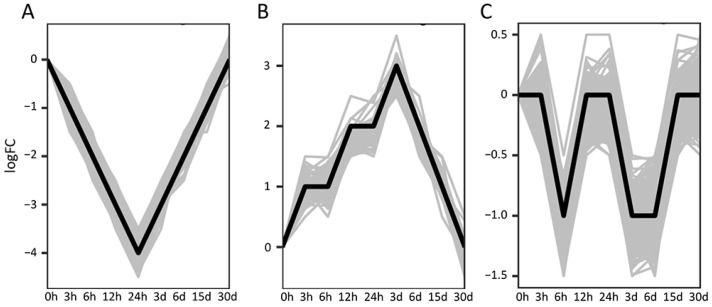
Three expression trends of DEGs in heart of *C. farreri* under heat stress. (**A**) Expression trend contrary to the trend observed in HR changes; (**B**) expression trend similar to the trend of HR; (**C**) expression trend opposite to the trend of HA changes. The *X*-axis represents the time of heat stress. The *Y*-axis represents the log_2_FC of gene expression change.

**Figure 5:**
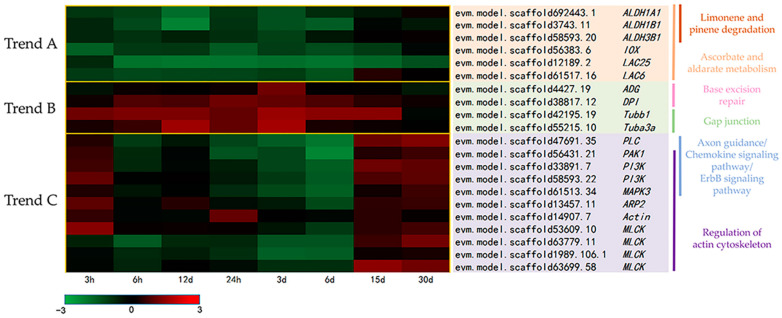
The expressions of key genes from three trends in heart of *C. farreri* under heat stress.

According to the differential analysis, a total of 3541 DEGs were identified, and the number of up-regulated genes and down-regulated genes in 3 h, 6 h, 12 h, 24 h, 3 d, 6 d, 15 d, and 30 d were 222, 126, 267, 232, 136, 51, 326, 299 and 503, 385, 429, 376, 1144, 1223, 82, 50, respectively (Figure 3). Under heat stress, the number of DEGs in the heart remained relatively stable within the first 24 h of heat stress. However, a significant increase was observed at 3 d and 6 d. Prior to 6 d, the number of downregulated genes substantially exceeded that of the upregulated ones. Beginning from 15 d of heat stress, there was a notable reduction in the number of DEGs, with an increase in the upregulated DEGs. After further categorization of the expression trends of 3541 DEGs, three distinct trends were identified, as illustrated in Figure 4. In expression trend A, a significant clustering of 711 DEGs was identified (*p* value < 0.05). This trend initially showed a decrease followed by an increase, which is contrary to the trend observed in heart rate changes. In expression trend B, another set of 106 DEGs exhibited an expression trend that was similar to the trend of heart rate, characterized by an initial increase followed by a decrease. However, the peak in this trend occurred with a little delay compared to the heart rate peak. Additionally, there was another distinct expression trend C, characterized by two fluctuations of initially decreasing and then increasing expression. This trend was significantly associated with 1188 DEGs (*p* value< 0.05). The expressions of key genes from three trends in hearts of *C. farreri* under heat stress are shown in Figure 5.

### 3.4. Functional Enrichment Analysis of DEGs

**Figure 6:**
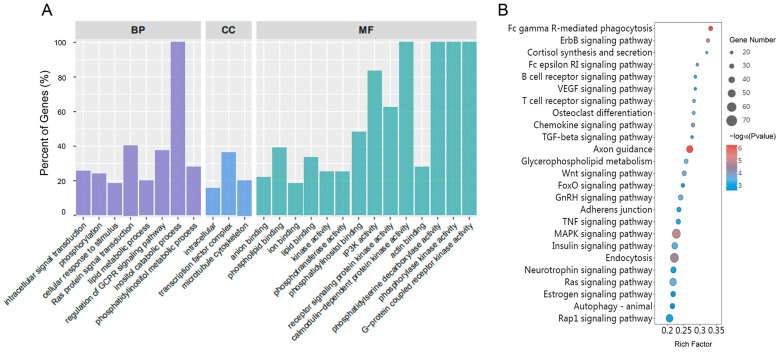
The GO and KEGG analysis of DEGs in heart of *C. farreri* under heat stress. (**A**) GO annotation. The *X*-axis represents the annotated functions. The *Y*-axis represents the percent of enriched gene numbers. (**B**) KEGG enrichment. The *X*-axis represents the rich factor of KEGG terms. The *Y*-axis represents the enriched pathways. The circle shape represents the number of DGEs in the enriched pathways.

The GO enrichment was used to classify the possible functions of the DEGs in the heart of *C. farreri*. There were 134, 21, and 113 terms enriched significantly (*p* value < 0.05) in Biological Process (BP), Cellular Component (CC), and Molecular Function (MF) (Table S3). Among them, the top 25 terms were shown in Figure 6A, including phospholipid binding (GO:0005543, MF), ion binding (GO:0043167, MF), lipid binding (GO:0008289, MF), calmodulin-dependent protein kinase activity (GO:0004683, MF), phosphatidylserine decarboxylase activity (GO:0004609, MF), phosphorylase kinase activity (GO:0004689, MF), G-protein coupled receptor kinase activity (GO:0004703, MF), intracellular signal transduction (GO:0035556, BP), inositol catabolic process (GO:0019310, BP), etc.

DEGs of the heart were enriched in 63 pathways significantly (*p* value < 0.05) according to the KEGG analysis (Table S4). These pathways could be classified into classes such as Metabolism, Environmental Information Processing, Cellular Processes, and Organismal Systems. Figure 6B lists the top 25 pathways, primarily related to the MAPK signaling pathway, FoxO signaling pathway, VEGF signaling pathway, phosphatidylinositol signaling system, the regulation of the actin cytoskeleton, etc.

### 3.5. Gene Coexpression Network

**Figure 7:**
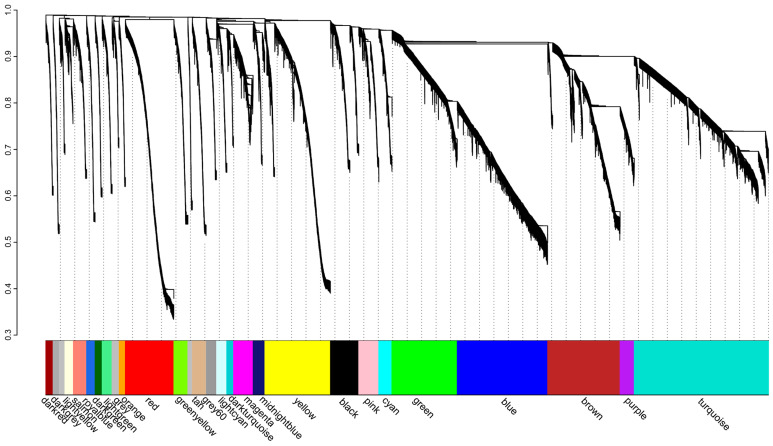
The WGCNA analysis of scallop heart transcriptome under heat stress. Dendrogram is produced by average linkage hierarchical clustering of genes based on topological overlap. Horizontal color bars represent different co-expression modules that are also numbered. Unassigned genes are labeled in grey.

**Figure 8:**
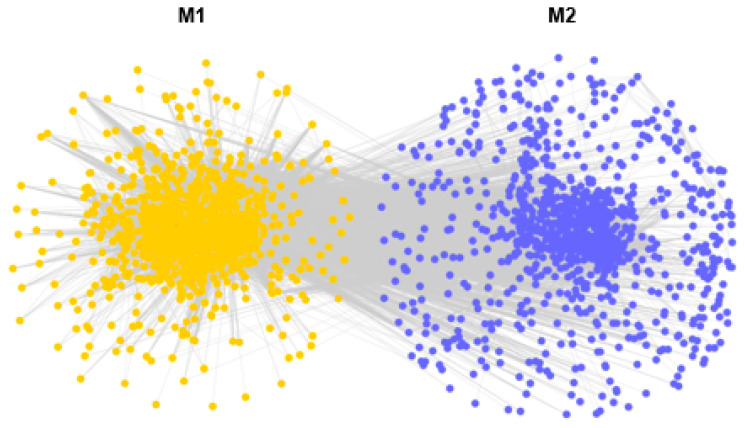
Main response modules of WGCNA network of heart after heat treatment. Yellow dots belong to M1 and blue dots belong to M2.

All RNA-seq data from 27 samples were used for calculation of gene-to-gene correlations. After eliminating the unexpressed genes, a total of 26 modules were derived from the expression data of 24,878 genes, with module size varying from 210 to 4620 (Figure 6; Table S5). The module yellow (M1) and module blue (M2) with FDR < 0.05 were identified as heat stress-responsive modules, which was shown in Figure 7 and Figure 8. There were 2251 genes in M1 and 3113 genes in M2. The number of DEGs were 163 in M1 and 478 in M2, respectively. GO analysis showed that genes in M1 were significantly enriched in membrane, transporter activity, and calcium ion binding (Table S6). Meanwhile, the possible functions of genes in M2 were motor activity, protein binding, and microtubule-based process (Table S7). KEGG analysis identified several common enrichments of pathways in both modules, including the calcium signaling pathway, vascular smooth muscle contraction, cGMP-PKG signaling pathway, and MAPK signaling pathway (Table S1).

### 3.6. Expression Patterns of Candidate Genes

**Figure 9:**
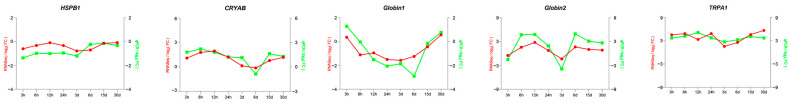
Quantitative real time-PCR validation. Green line represents RT-qPCR results; red line represents RNA-Seq results.

To verify the accuracy of transcriptome sequence data, a total of five genes, Heat shock protein beta-1 (*HSPB1*), Crystallin alpha B (*CRYAB*), Globin1, Globin2, and Transient receptor potential cation channel subfamily A member 1 (*TRPA1*) were randomly selected for RT-qPCR (Figure 9). RT-qPCR results (green line) of these genes were consistent with RNA-Seq results (red line), indicating the high reliability of the transcriptome.

## 4. Discussion

The heart plays a crucial role in the circulatory system by facilitating the transport of gases and nutrients, as well as maintaining homeostasis and supporting various physiological functions throughout different conditions and stages of life [47,48]. An increase in heart rate plays a positive role in marine animals’ response to stressful environments, such as high temperatures and low oxygen. In this study, when Zhikong scallops *C. farreri* were exposed to heat stress, significant increase in their heart rate were observed. The increase of heart rate might be a physiological regulation behavior of scallops to cope with acute heat stress by adjusting the supply of oxygen and substances. Heart rate is an important indicator for measuring cardiac functionality [19,20,21]. And the subsequent gradual recovery of heart rate indicates that scallops can adapt to the temperature condition of 27 °C and eventually tend to homeostasis. Similar phenomena of heart rate variation have also been reported in other mollusks and heart rate-related indicators such as ABT (Arrhenius break temperature) have been used in many studies to assess individual stress tolerance [26,49]. For example, the ABT values of two warm water species (*A. irradians* and *A. ventricosus*) were found to be significantly higher than those of two cold water species (*C. farreri* and *P. yessoensis*), which indicated that ABT could serve as a reliable indicator of the thermal tolerance for scallops [49]. In abalone, the heart rates exhibited a sharp decline below a certain dissolved oxygen threshold, which can be referred to as the breakpoint of dissolved oxygen (BPDO). Further analysis showed that BPDO was significant related with the survival time of individuals under hypoxia (~0.5 mg/L) and can be used to select hypoxic-tolerant individuals for further genetic breeding [50]. These studies suggest that heart rate and the related indicators are important in studying stress and tolerance in mollusks. Regarding the heart amplitude results, although there were no significant changes under high-temperature stress, a fluctuating trend was observed. Notably, during acute heat stress before 12 h, an inverted V-shaped change was evident, a trend that has also been found in the heat stress of the bay scallop (*A. irradians*). The inverted “V” trend implied that the homeostasis of Zhikong scallops with heat stress may be maintained by increasing the oxygen absorption to enhance oxygen supply, during which the metabolic changes in poikilotherms have been reported to be linked to higher temperature adaptation. According to the results of HA in this study, the changes continued to show an inverted V-shaped trend during the subsequent 30-day period. Moreover, we found that HA increased with duration of high-temperature stress and showed a certain recovery trend at the end. This suggested that under long-term stress, the heart may adapt to high temperatures by regulating its amplitude. This may be that under long-term heat stress, the scallops gradually adapt to the high-temperature stress, thus reducing the demand for the large supply of oxygen, so their hearts did not need to maintain a high level of amplitude.

Furthermore, the antioxidant capacities of the heart were measured to investigate the physiological responses of Zhikong scallops under heat stress. Two essential antioxidant enzymes, SOD and CAT, were reported to cooperatively ameliorate organisms’ damages resulting from heat stress by scavenging accumulated ROS (reactive oxygen species). Consistent with such similar synergistic alterations in SOD and CAT activities in other mollusks facing thermal threats, our findings also showed significant upregulation of SOD and CAT, and this protective reaction might prevent the possible damage of scallops from heat stress.

Transcriptomics are often used to explore the molecular mechanism behind biological progress [51]. According to the heart transcriptomes analysis, under heat stress, the number of DEGs over a 30-day stress period exhibited a trend of initially increasing and then decreasing. The DEGs were further classified into three different trends, indicating different regulatory mechanisms in the heart under heat stress.

Pathway enrichment analysis showed that in expression trend A, 711 differentially expressed genes were significantly enriched in pathways such as limonene and pinene degradation, ascorbate and aldarate metabolism, and fatty acid degradation (Table S8a). Limonene and pinene degradation were the most significant pathway (*p* value < 0.05). There were three DEGs enriched in this pathway: *ALDH1A1* (aldehyde dehydrogenase family 1 member A1, evm.model.scaffold692443.1), *ALDH1B1* (aldehyde dehydrogenase family 1 member B1, evm.model.scaffold3743.11), and *ALDH3B1* (aldehyde dehydrogenase family 3 member B1, evm.model.scaffold58593.20), all belonging to the aldehyde dehydrogenase (*ALDH*) family and were down regulated before 3 d (Figure 5). Therefore, we speculated that this pathway was involved in the cardiac response of scallops to high temperature by regulating the antioxidant capacity of ALDH. Moreover, these three genes were also enriched in the ascorbate and aldarate metabolism. In vertebrates, aldehyde dehydrogenase is an important enzyme involved in the detoxification of aldehydes and the mitigation of oxidative stress by converting toxic lipid peroxides, such as aldehydes, into non-toxic acids [52,53]. So, aldehyde dehydrogenase helps prevent the accumulation of toxic aldehyde compounds, thereby protecting cells from oxidative stress and contributing to overall cellular health and longevity [54,55,56]. In *C. farreri*, down-regulation of *ALDH*s was found under heat stress, which indicated a weakened function of this protection in the heart under heat stress. Conversely, the enzyme activity assays indicated a significant increase in SOD and CAT activities within the first 3 h, suggesting that the primary antioxidant mechanisms in scallops in response to heat stress are activated during this period. Consequently, the total antioxidant capacity remains stable within this timeframe. In addition, other oxidases in the ascorbate and aldarate metabolism pathway also showed a downward trend before 6 d, such as inositol oxygenase (*IOX*, evm.model.scaffold56383.6) and L-ascorbate oxidase (*LAC25*, evm.model.scaffold12189.2; LAC6, evm.model.scaffold61517.16). In *Drosophila melanogaster*, inositol oxygenase catalyzes the conversion of myo-inositol to glucuronic acid, which is a key step in inositol catabolism [57]. The down-regulation of *IOX* in scallops under high temperatures may suggest that heat stress inhibits inositol catabolism. Finally, L-ascorbate oxidase is involved in the oxidation of L-ascorbic acid to dehydroascorbic acid, thus it is crucial in maintaining the redox balance within cells [58,59]. As L-ascorbic acid can effectively remove free radicals in the body and reduce the damage of free radicals to cells, thereby protecting cells from oxidative stress, so the down-regulation of its oxidase was conducive to maintaining the function of the heart of *C. farreri* in high-temperature environments. Overall, in the heart of *C. farreri* under heat stress, the expression levels of these oxidases decreased, whereas the activities of antioxidant enzymes such as SOD and CAT increased. Concurrently, heart rate increased before 6 d. These observations suggest that an enhanced antioxidant capacity may play a crucial role in maintaining cardiac physiology, thereby promoting survival and adaptability to heat stress conditions.

In expression trends B, 106 differentially expressed genes were predominantly involved in significant pathways like base excision repair, gap junction, chloroalkane, and chloroalkene degradation, among which base excision repair was the most significant (*p* value < 0.05) (Table S8b). A/G-specific adenine DNA glycosylase (*ADG*, evm.model.scaffold4427.19) and DNA polymerase I (*DPI*, evm.model.scaffold38817.12) were enriched in the base excision repair pathway and were up-regulated during the heat stress (Figure 5). A/G-specific adenine DNA glycosylase is an enzyme that plays a crucial role in the base excision repair (BER) pathway, which is responsible for correcting damaged or inappropriate bases in DNA [60,61]. In an experimental heart failure (HF) model in rats, similar to our results, significant up-regulation of DNA glycosylase activities was observed, and this up-regulation initiated the BER pathway, which could play an important role during HF by counteracting the effect of genotoxic stress, structural damage of tissue, and myocardial remodeling [62]. Our results showed that two of the key enzymes, A/G-specific adenine DNA glycosylase and DNA polymerase I, were up-regulated in the BER process, which enhanced the correction of damaged or inappropriate bases in DNA to resist heat stress in the heart of *C. farreri* under heat stress. In the gap junction pathway, tubulin alpha-3 (*Tuba3a*, evm.model.scaffold55215.10) and tubulin beta-1 (*Tubb1*, evm.model.scaffold42195.19) were up-regulated. They are both critical components of microtubules, which work together to form microtubules through dynamic polymerization and depolymerization processes driven by the binding and hydrolysis of GTP (guanosine triphosphate) [61,62]. Under heat stress, the up-regulation of these genes in the scallop heart may contribute to the maintenance of cellular shape and integrity, intracellular transport, and cell division.

The expression trend C showed two fluctuations of decreasing and increasing, in which axon guidance, chemokine signaling pathway, and ErbB signaling pathway was most significantly enriched (*p* value < 0.05) (Table S8c). Five genes were enriched in all three pathways: Mitogen-activated protein kinase 3 (*MAPK3*, evm.model.scaffold61513.34), p21-activated kinase 1 (*PAK1*, evm.model.scaffold56431.21), Phosphatidylinositol-4,5-bisphosphate 3 kinase (*PI3K*, evm.model.scaffold33891.7; evm.model.scaffold58593.22), and 1-phosphatidylinositol-4,5-bisphosphate phosphodiesterase (*PLC*, evm.model.scaffold47691.35). Mitogen-activated protein kinases (*MAPK*) comprise a family of ubiquitous proline-directed, serine/threonine-protein kinases, which lie in protein kinase cascades and participate in signal transduction pathways that could control intracellular events including acute responses to environmental stress [63,64].

P21-activated kinases (*PAK*) are key effectors of the small GTPases *Rac1* and *Cdc42*, as well as Src family kinases. *PAK1* has several well-documented roles such as cell proliferation, adhesion, and migration [65]. In particular, *PAK1* has been founded in cardiac physiology and cardio protection. For example, *PAK1* roles in the regulation of excitability and contractility of the heart [66,67,68]. *PAK1* was identified in cardiomyocyte Ca^2+^ handling, and it was demonstrated that it functions through unique mechanisms involving regulation of the post-transcriptional activity of key Ca^2+^-handling proteins, including the expression of Ca^2+^-ATPase [69]. In this study, under heat stress, expression of *MAPK3* and *PAK1* in scallop hearts showed two fluctuations of decreasing and increasing, which may regulate cardiac excitability and contractility, thus influencing the heart amplitudes that showed a contrary expression trend. *PI3K* is a critical player in the signal transduction pathways initiated by growth factors, hormones, and other extracellular stimuli. It converts phosphatidylinositol-4,5-bisphosphate (*PIP2*) into phosphatidylinositol-3,4,5-trisphosphate (*PIP3*) [70,71]. While *PLC* hydrolyzes *PIP2* to generate two important secondary messengers: inositol-1,4,5-trisphosphate (*IP3*) and diacylglycerol (*DAG*) [72]. IP3 binds to IP3 receptors (*IP3R*) on the endoplasmic reticulum, reducing the release of calcium ions from the ER into the cytoplasm [73,74]. The elevation of intracellular free calcium ion concentration may modulate cardiac contraction, corresponding to heart rate variation in *C. farreri* under heat stress.

WGCNA analysis was applied to classify the possible functions of the expressed genes, which may drive *C. farreri* response to heat stress. Although the genes were divided into two modules, Figure 8 showed a close relationship between them. KEGG pathway analysis revealed that these modules shared overlapping pathways, including calcium signaling pathway, vascular smooth muscle contraction, cGMP-PKG signaling pathway, and MAPK signaling pathway, as shown in Tables S6 and S7.

The calcium signaling pathway was widely related to signal transduction as well as environmental stress responses of organisms [75,76,77]. G protein-coupled receptors (*GPCR*s) are a class of cell surface receptors that regulate various signaling pathways inside cells by responding to external signals [78,79,80]. *GPCR*s are involved in the calcium signaling pathway and can regulate the concentration of calcium ions in the cell. A typical example is that certain *GPCR*s can activate G proteins to initiate the hydrolysis of phosphatidylinositol-4,5-diphosphate (*PIP2*), so that inositol-1,4,5-triphosphate (*IP3*) is produced [80]. In this study, after exposure to heat stress, *GPCR* (M1-evm.model.scaffold42667.6, M1-evm.model.scaffold48689.2, M2-evm.model.scaffold35009.3, M2-evm.model.scaffold40853.21.1) in the calcium signaling pathway was significantly down-regulated in the heart of *C. farreri* after exposure to heat stress, which may lead to the decrease of intracellular *IP3*. And at the same time, the *IP3R* genes (M1-evm.model.scaffold62249.5, M2-evm.model.scaffold63833.21, M2-evm.model.scaffold726937.1, M2-evm.model.scaffold61517.13) of the heart showed a sustained down-regulated expression trend, which may reduce the accumulation of calcium ions in the ER and at the same time increase the release of calcium ions from the ER into the cytoplasm, ultimately leading to an increase in intracellular levels of free calcium ions. Therefore, the calcium signaling pathway regulated the concentration of intracellular free calcium ions, and scallops may respond to heat stress by regulating intracellular calcium ion concentration.

Furthermore, regulation of actin cytoskeleton was specifically enriched (*p* value < 0.05) in M2 (Table S7). In this pathway, 34 DEGs were enriched, such as actin (evm.model.scaffold14907.7), actin-related protein 2 (*ARP2*, evm.model.scaffold13457.11), myosin light chain kinase (*MLCK*, evm.model.scaffold53609.10, evm.model.scaffold63779.11, evm.model.scaffold1989.106.1, evm.model.scaffold63699.58), and myosin light chain phosphatase (*MLCP*, evm.model.scaffold55531.13). In the heart, actin is a key cytoskeletal protein involved in the contraction and relaxation of cardiomyocytes. It regulates the movement of cardiomyocytes by forming myosin complexes with myosin and troponin [81]. *ARP2* participates in regulating cytoskeletal recombination and cell motility in cardiomyocytes. Together with actin, it plays a role in the contraction and relaxation of cardiomyocytes [82,83]. *MLCK* and *MLCP* regulates myosin light chain (*MLC*) phosphorylation, which is determined by the balance of its phosphorylation and dephosphorylation activities and plays important roles in regulating the contraction process of cardiomyocytes [84,85,86]. These regulatory factors influence the contractility and elasticity of cardiomyocytes, consequently impacting the amplitude of cardiac activity, which exhibited a biphasic trend characterized by an initial increase followed by a decrease. This observation suggests that regulation of protein phosphorylation plays a critical role in the heart of scallops responding to heat stress.

## 5. Conclusions

In the present study, we investigated the potential mechanisms underlying the cardiac response to heat stress in *C. farreri*. Our finding indicated that high temperature significantly affects the cardiac function of Zhikong scallops, which was proved by both heart rate and antioxidant capacity of *C. farreri* initially increased and subsequently returned to baseline levels at the end of 30-day heat stress period. The down-regulation of oxidases, coupled with the up-regulation of antioxidant enzymes such as SOD and CAT, in the heart of *C. farreri* proved that antioxidant mechanisms are essential for maintaining cardiac function and promoting survival under heat stress. Additionally, up-regulation of key enzymes involved in the base excision repair and gap junction pathways suggested that the heart of *C. farreri* under heat stress enhanced DNA repair and microtubule integrity to preserve cellular structure and function. Furthermore, the variable expression of key signaling molecules and cytoskeletal regulators suggested that the calcium signaling pathway, vascular smooth muscle contraction, cGMP-PKG signaling pathway, and MAPK signaling pathway played roles in modulating cardiomyocyte contraction in *C. farreri* under heat stress. Collectively, our findings provide novel insights into the cardiac response of the scallops to heat stress and enhance our understanding of potential adaptative mechanisms in scallops in the context of global warming.

## Data Availability

The data presented in this study are available in the article.

## Supplementary Materials

The following supporting information can be downloaded at: https://www.mdpi.com/article/10.3390/antiox13101217/s1, Table S1: Primers for quantitative real time-PCR validation; Table S2: Summary of RNA-Seq sequencing information in heart of *C. farreri* after heat stress; Table S3: GO analysis of DEGs; Table S4: KEGG analysis of DEGs; Table S5: FDR of modules and module size by WGCNA analysis in *C. farreri* heart after heat stress; Table S6: GO and KEGG analysis of M1; Table S7: GO and KEGG analysis of M2; Table S8: KEGG analysis of three expression trend.
